# Reconstructed human intestinal comet assay, a possible alternative *in vitro* model for genotoxicity assessment

**DOI:** 10.1093/mutage/gead011

**Published:** 2023-04-28

**Authors:** Christopher Owen Hughes, Hui Kheng Lim, Joseph Choon Wee Tan, David Ian Leavesley, Benjamin Paul Chapman Smith

**Affiliations:** Innovations in Food and Chemical Safety (IFCS) Programme, Agency for Science, Technology and Research, Singapore, Singapore; Skin Research Institute of Singapore (SRIS), Agency for Science, Technology and Research, Singapore, Singapore; Future Ready Food Safety Hub (a joint initiative of A*STAR, SFA & NTU), Nanyang Technological University, Singapore, Singapore; Innovations in Food and Chemical Safety (IFCS) Programme, Agency for Science, Technology and Research, Singapore, Singapore; Skin Research Institute of Singapore (SRIS), Agency for Science, Technology and Research, Singapore, Singapore; Future Ready Food Safety Hub (a joint initiative of A*STAR, SFA & NTU), Nanyang Technological University, Singapore, Singapore; Future Ready Food Safety Hub (a joint initiative of A*STAR, SFA & NTU), Nanyang Technological University, Singapore, Singapore; Innovations in Food and Chemical Safety (IFCS) Programme, Agency for Science, Technology and Research, Singapore, Singapore; Skin Research Institute of Singapore (SRIS), Agency for Science, Technology and Research, Singapore, Singapore; Innovations in Food and Chemical Safety (IFCS) Programme, Agency for Science, Technology and Research, Singapore, Singapore; Future Ready Food Safety Hub (a joint initiative of A*STAR, SFA & NTU), Nanyang Technological University, Singapore, Singapore; Singapore Institute of Food and Biotechnology Innovation (SIFBI), Agency for Science, Technology and Research, Singapore, Singapore

## Abstract

The aim of the present study was to evaluate the compatibility of reconstructed 3D human small intestinal microtissues to perform the *in vitro* comet assay. The comet assay is a common follow-up genotoxicity test to confirm or supplement other genotoxicity data. Technically, it can be performed utilizing a range of *in vitro* and *in vivo* assay systems. Here, we have developed a new reconstructed human intestinal comet (RICom) assay protocol for the assessment of orally ingested materials. The human intestine is a major site of food digestion and adsorption, first-pass metabolism as well as an early site of toxicant first contact and thus is a key site for evaluation. Reconstructed intestinal tissues were dosed with eight test chemicals: ethyl methanesulfonate (EMS), ethyl nitrosourea (ENU), phenformin hydrochloride (Phen HCl), benzo[a]pyrene (BaP), 1,2-dimethylhydrazine hydrochloride (DMH), potassium bromate (KBr), glycidamide (GA), and etoposide (Etop) over a span of 48 h. The RICom assay correctly identified the genotoxicity of EMS, ENU, KBr, and GA. Phen HCl, a known non-genotoxin, did not induce DNA damage in the 3D reconstructed intestinal tissues whilst showing high cytotoxicity as assessed by the assay. The 3D reconstructed intestinal tissues possess sufficient metabolic competency for the successful detection of genotoxicity elicited by BaP, without the use of an exogenous metabolic system. In contrast, DMH, a chemical that requires liver metabolism to exert genotoxicity, did not induce detectable DNA damage in the 3D reconstructed intestinal tissue system. The genotoxicity of Etop, which is dependent on cellular proliferation, was also undetectable. These results suggest the RICom assay protocol is a promising tool for further investigation and safety assessment of novel ingested materials. We recommend that further work will broaden the scope of the 3D reconstructed intestinal tissue comet assay and facilitate broader analyses of genotoxic compounds having more varied modes of actions.

## Introduction

The assessment of genotoxic potential of pharmaceuticals, agrochemicals, novel foodstuffs, additives, and other materials includes a battery of assays that evaluate the three main types of genotoxic insult: gene mutation, clastogenicity, and aneugenicity. *In vitro* assays such as Ames reverse mutation, micronucleus and mammalian forward mutation assays [1–3] are corroborated using *in vivo* micronucleus, comet, and rodent assay protocols [4–6]. However, concerns persist with the specificity of *in vitro* assay protocols, particularly the incidence of false positive results [7] which may necessitate additional *in vivo* assessment. This has to some degree been countered through the refinement of existing protocols [8,9] and with a shift away from cell lines such as CHO, to p53-competent cells such as TK6, or *ex vivo* human lymphocytes [10]. Notwithstanding, regulatory bodies continue to require *in vivo* genotoxicity testing, which is performed [11–13], typically, in rodent models.

Seeking to improve the relevance, specificity, and mechanistic insight, and provide greater relevance to actual human exposure, genotoxicity measures more indicative of genuine *in vivo* risk for humans, and community pressure to replace, reduce, and refine protocols that include animals, several new *in vitro* technologies have been developed to provide higher levels of confidence in specificity, mechanisms of toxicity, and relevance for humans [14–17]. The cosmetics industry has enabled many recent advances, for example, the incorporation of 3D skin tissue models both for micronucleus and comet assay protocols [14,15,18–21].

The comet assay, also known as Single Cell Gel Electrophoresis, is a simple genotoxicity assay used for the detection of DNA damage. The assay is sensitive to single- and/or double-strand DNA breaks and other lesions that may give rise to genetic mutations. In brief, individual cells are suspended in agar, lysed, and then subjected to electrophoresis in alkaline conditions. Damaged and fragmented DNA is separated from the main nucleoid body under the electromagnetic field and forms tailed comet-like structures that can then be stained and visualised. The endpoint of the comet assay is a qualitative indicator of damage rather than a definitive measure of unrepaired DNA damage, such as reported by the micronucleus test [22–24]. Currently, the comet assay forms part of the *in vivo* genotoxicity testing battery [5], however, a defined *in vitro* approach is lacking. Efforts have been dedicated to standardizing and validating the *in vitro* comet assay in different cell lines [25,26] as well as in 3D skin models [15]. As far as we are aware, no *in vitro* protocol has been developed for intestinal tissues and the oral route of exposure.

The main attractions of the comet assay protocol as a measure of genotoxicity are that it is technically undemanding, requires only a small number of cells per sample, is easily applied to many tissue and cell types (proliferating and nonproliferating), and, importantly, yields high sensitivity to some types of genotoxins. Nevertheless, the comet assay is not without limitations such as sensitivity to damage induced by aneugens and DNA crosslinkers which are not readily detected in the basic alkaline assay [27,28]. Whilst *in vivo* assay results are typically ascribed greater confidence than *in vitro* assay results, concerns do also exist that the incidence of false positive results coincide with high tissue-specific toxicity, or histopathological changes [29]. The comet assay has the advantage that it may be combined with other assay protocols in a single study, or animal, with appropriate treatment regimens. Accepting this, most *in vivo* comet testing is performed in rodents [11–13,30]; an animal model increasingly acknowledged to offer lesser physiological relevance for humans. Our particular assay protocol was developed and evaluated as an extension of our recently published micronuclei cytome (RICyt) assay protocol [31].

This study describes our proof of compatibility of a comet assay protocol with the commercially available reconstructed human intestinal model (EpiIntestinal™, MatTek). Our intention was to evaluate the applicability of the EpiIntestinal™ platform as another protocol in the genotoxicity toolbox, one that might be used as a preview of the small intestine *in vivo* comet assay. We investigated a total of eight well-established validation test chemicals. Assay acceptance criteria have been proposed in this manuscript. We have named the assay ‘Reconstructed Intestine Comet (RICom) Assay’.

## Materials and methods

### Chemicals and other materials

Reference chemicals benzo[a]pyrene (BaP, B1760), ethyl methanesulfonate (EMS, M0880), 1,2-dimethylhydrazine hydrochloride (DMH, D161802), etoposide (Etop, E1383), phenformin hydrochloride (Phen HCl, PHR1573), potassium bromate (KBr, 309087), glycidamide (GA, 04704), and ethyl nitrosourea (ENU, N3385) were purchased from Sigma Aldrich (St. Louis, MO). Test chemical stock solutions were prepared in anhydrous DMSO (Sigma Aldrich, St. Louis, MO) or in water.

Cell recovery solution (Corning, Tewksbury, MA) and human tumour dissociation kit (130-095-929, Miltenyi Biotec, Bergisch Gladbach, Germany) were used for the purpose of 3D tissue dissociation as previously described [31].

Comet assay kit consisting of low-melt agarose (4250-050-02, 1% low melting agarose in PBS), cell lysis solution (4250-050-01), pre-coated 2-gel agarose comet slides (CometSlide™ 4250-200-03), and electrophoresis apparatus (4250-050-ES) were sourced from RnD systems (Minneapolis, MN). Low-melt agarose was reused up to 10 times after melting. TE buffer was obtained from Promega (Madison, WI). Alkali DNA unwinding (300 mM NaOH, 1 mM EDTA) and electrophoresis solutions (200 mM NaOH, 1 mM EDTA) were prepared fresh for use on the day of experiment. SYBR Green DNA stain was procured from Thermo Fisher Scientific (Waltham, MA).

### 3D tissue culture

EpiIntestinal™ tissues (SMI-200-FT v2.0) and culture media (SMI-100-MM) were purchased from MatTek Corporation® (Ashland, MA). The microtissue model is cultured on Transwell™ multiple well plate consisting of permeable membrane inserts, which create upward facing apical compartments and basolateral compartments at the receiving well. Tissues are shipped in 24-well agar-coated plates. Upon arrival, the tissues were revived in SMI-100-MM and maintained at 37°C, 5% CO_2_/95% air, and 98% relative humidity for a minimum of 1–4 days prior to exposure to test materials and commencement of the assay.

### Experimental design

**Figure 1.**
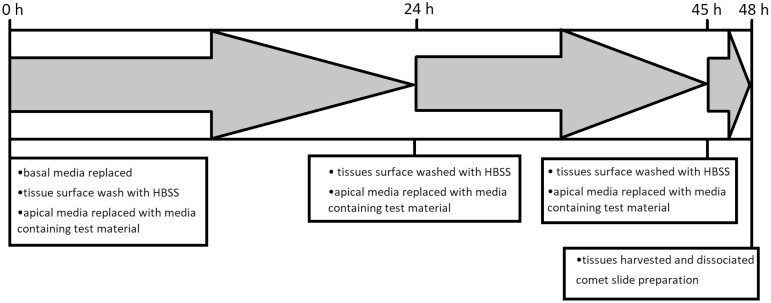
RICom assay treatment timeline of EpiIntestinal™ microtissues. The test chemicals are administered three times: the second administration is 24 h after the first administration. The last administration is 21 h after the second administration (at 3 h before tissue harvesting). Tissues are exposed to test chemicals for 48 h in total.

After revival, the apical surface of the tissues was gently rinsed with Hanks Balanced Salts Solution containing calcium and magnesium (HBSS +Ca/Mg). The apical media is replaced with fresh SMI-100-MM media containing test materials of interest dissolved in solvent (i.e. 1% DMSO or 10% water). Tissues were incubated for 24 h (as above), again rinsed with HBSS +Ca/Mg, and fresh media containing test material was applied to the apical compartment. Twenty-one hours after the second exposure, tissues are rinsed, and dosed again for a final 3 h before harvesting and subject to comet assay protocol (below). The complete treatment procedure requires 48 h (Fig. 1). The treatment schedule mimics that of other comet assay protocols, where animals/tissues are treated over a 2-day period to ensure metabolism and maximal exposure, with a final treatment period a few hours prior to tissue harvest to capture any short-duration DNA lesions [5,32]. The results for the comet assay were generated from three independent experiments. Each experiment consisted of a single microtissue per test concentration.

### Tissue dissociation

Tissue dissociation was performed as previously described in Lim *et al.* [31] where intact cells were fixed and examined under a microscope. Transwell™ inserts were removed and washed with pre-warmed HBSS +Ca/Mg to remove cell debris, remaining culture media and test material. The culture membranes with tissues were then excised from the insert and incubated with pre-warmed Cell Recovery solution (Corning, Tewksbury, MA) for a maximum of 15 min to aid whole tissue detachment from the collagen coated cell culture inserts. The mixture was pulse-agitated using a vortex mixer at 5 min intervals to promote tissue detachment from the Transwell™ membrane. Remnant tissue can be detached fully from the culture membrane with the use of forceps. Detached tissues recovered into in cell recovery solution were placed into gentleMACS C-tubes (Miltenyi Biotech, Bergisch Gladbach, Germany) containing the enzyme mix described in the human tumour dissociation kit’s protocol. The tubes were then transferred into a gentle MACS octo dissociator with heater (Miltenyi Biotech, Bergisch Gladbach, Germany), and subjected to one cycle of ‘37C_m_LPDK_1’ program (pre-installed manufacturer program). Dissociated cells were resuspended in 1 ml of ice-cold HBSS without Ca/Mg for total cell enumeration using a haemocytometer. Total handling time per batch of tissues from the end of treatment to tissue washing and dissociation, cell count, and initiation of comet assay procedure preparation of was <1 h.

### Alkaline comet assay procedure

The Alkaline Comet assay was performed using comet assay kit (4250-050-ESK, Trevigen, Gaithersburg, MD) according to the manufacturer’s protocols, and modified procedures published by Singh *et al.* [33] Fifteen microlitres of isolated cells at 100,000 cells/ml in HBSS without Ca/Mg, were mixed with 135 µl LMA (melted and pre-warmed at 37°C, final agarose contest 0.9%). About 50 µl of cells in LMA then were immediately transferred to each of the two spots on agarose pre-coated glass slides. Duplicate slides were prepared for each tissue. Slides were cooled at 4°C in the dark for 30 min to allow LMA to solidify. Slides were then incubated at 4°C for a minimum of 1 h with pre-chilled cell lysis buffer. Slides were then incubated in alkali DNA unwinding solution at room temperature, for 20 min. Slides were placed in an electrophoresis chamber filled with 850 ml electrophoresis buffer and pre-chilled to 4°C, subjected to 21 V direct current (1 V.cm^−1^) for 30 min. After electrophoresis, slides were immediately neutralized in distilled water, rinsed twice further for 10 min, before being dehydrated in 70% ethanol for 5 min. Slides were then left to dry overnight at room temperature. For DNA staining, 100 µl of 1× SYBR Green in TE buffer was added to each slide spot and left for 45 min at 4°C. Excess stain was removed, and slides were allowed to dry in the dark before microscopic analysis.

### Slide analysis

Samples were scored and analysed using the Metafer 4 v3.13.3 scoring platform (Metasystems, Germany). DNA damage was measured and reported as the median % tail DNA per microtissue.

### Assay acceptance criteria

Proposed acceptance criteria for RICom assay in this paper:

1. Experiment should include solvent control, positive control, and at least three concentrations of the test articles.
2. At least one microtissue per test condition per experiment should be included. The final results should be generated from three independent experiments for this experiment to investigate inter-assay variability.
3. Minimum of 100 comets scored per tissue.
4. The mean %tail DNA for solvent control must be within the established upper historical negative range (≤5.44% in this study). Historical range is defined as historical mean ± 2 standard deviations.
5. A positive result is defined when test chemicals show a statistically significant increase in %tail DNA in at least one test concentration that is also above the established historical negative range of solvent control (5.44%).
6. In the event of excessive cytotoxicity (≥50% reduction in cell number relative to the solvent control), the sample should be excluded from evaluation. For the *in vitro* comet assay, there has not been an established threshold or method of assessment for cytotoxicity, with some publications and methods varying from 20% to 50% cytotoxicity [34]. The authors for this study chose an upper limit; as further data are produced, this could be refined empirically.
7. Hedgehog comets, comets with almost all their DNA in the tail and minimal/no head are excluded from %tail DNA data analysis [5].
8. Solvent control tissues must contain a minimum of 1,000,000 cells per tissue, lower values may be indicative of an error during tissue dissociation or tissue viability.

### Statistical analysis

All data are presented as means ± standard deviation of median or mean as indicated in the figure legend. Two-tailed one-way Anova and Tukey’s *post-hoc* test was applied to determine any %tail DNA significance between test concentration and solvent control groups. Data were deemed significant if *P* < 0.05. All experiments were performed three times independently. Each experiment consisted of a single microtissue per test condition.

## Results and discussion

### Relevance of the Reconstructed Intestine Comet (RICom) assay for hazard identification

The aim of this study was to assess the feasibility of running the *in vitro* comet assay in reconstructed human 3D small intestine microtissues. Specifically, we adapted a comet assay protocol (RICom) for use with reconstructed 3D EpiIntestinal™ microtissues (MatTek Corporation) and demonstrated that this protocol offers a suitable technology platform for identifying ingested genotoxic hazards for human ­intestinal tissues. The RICom assay protocol is informed by the well-established 3D Skin Comet assay published by Pfuhler *et al.* [15], OECD *in vivo* comet guideline [5], and the recently published RICyt micronucleus test [31]. The 3D EpiIntestinal™ model is a reconstructed mimic of human small intestine tissue that retains native tissue architecture (microvilli), ­polarization (basement membrane, villin), heterogenous cell populations (enterocytes, paneth cells, M cells, tuft cells, intestinal stem cells), barrier function, and biochemical activity akin to native human intestinal tissue [16]. Though not identical to the human gut, the 3D EpiIntestinal™ model offers the potential of a more realistic model over current 2D *in vitro* systems. With further validation work, the RICom could be used as a bridging model between *in vitro* 2D and *in vivo* comet assay for genotoxic hazard identification through the intestinal route of exposure. The RICom assay complements the recently developed Reconstructed Intestinal Micronuclei Cytome (RICyt) assay [31].

### Historical negative range data generation

**Figure 2.**
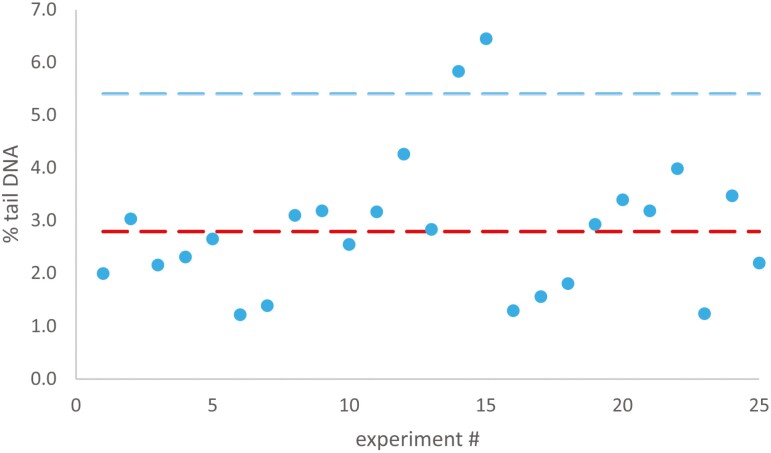
Historical negative range for solvent control used in RICom assay. Percentage of tail DNA (blue dots) in individual EpiIntestinal™ microtissues treated with solvent control (1% DMSO or 10% H_2_O). Each dot represents data from a single treated tissue. Lower red-dashed line denotes historical mean value. Upper blue-dashed line denotes two standard deviations upper limit. Data are reported as median % tail DNA.

A clear comet positive result must exceed the historical negative range in at least one test concentration in addition to statistical significance. Historical range for this assay being defined as two standard deviations within the historical mean [35]. A low background %tail DNA will also allow for greater sensitivity to small changes in DNA damage and assessment of routine assay performance. Here, the assay was performed using solvent control-treated (1% DMSO or 10% water) tissues. Our intent was to acquire and determine the basal range of DNA damage, as well as to determine variability between experiments. Laboratory-specific historical reference for solvent control can be derived and used for subsequent data analysis. The EpiIntestinal™ tissues treated with solvent controls expressed average background tail DNA of 2.8% with a two standard deviation upper limit of 5.44% (*n* = 25). Historical negative data range was generated during assay validation and test chemical assessment (see Fig. 2). On occasions where solvent/negative controls exceeded the two standard deviation range and no technical errors were noted, data were collected for use in historical negative data generation, but data were not used for assessment of validation chemicals.

### Ethyl methanesulfonate, EMS (CAS 62-50-0)

**Figure 3.**
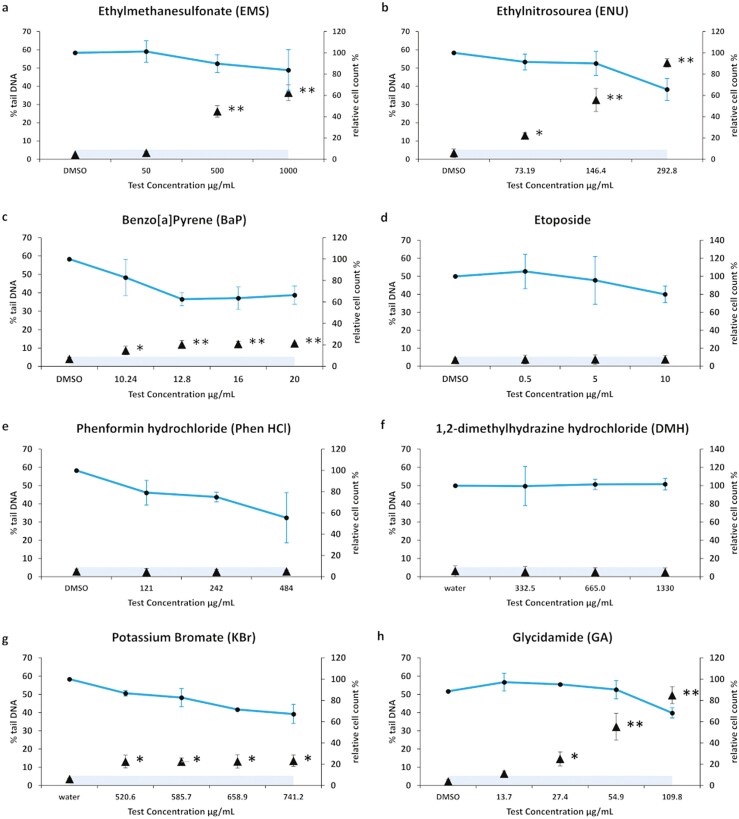
RICom assay evaluates DNA damage induction in EpiIntestinal™ tissue. The %tail DNA (black-filled triangle) for the 3D EpiIntestinal™ tissue is given as mean ± standard deviation. The shaded areas indicate the reference range (historical negative range) for solvent controls. Solid line denotes relative cell count (%). The results from three independent experiments are shown. Each experiment consisted of a single microtissue per test condition. Data for DNA damage are reported as mean of median %tail DNA. Data for cytotoxicity are reported as mean of mean relative cell count. *P < 0.05 and **P < 0.01.

EMS is commonly used as positive control for genotoxicity assays [36], including *in vivo* comet in many tissues/organs, such as the upper intestine [36,37]. EMS alkylates DNA, leading to point mutations, single-stranded breaks, and chromosomal aberrations [38]. Multiple treatment groups produced a statistically significant increase in % tail DNA relative to the solvent control, a %tail DNA that was also above historical negative values. We accept that this represents a clear positive measure. The maximum test concentration was selected by the disappearance of scorable comets (excess hedgehog comets), or threshold of cytotoxicity described in score acceptance criteria, above. The maximum test concentration for EMS is, therefore, limited to 1000 µg/ml (8.05 mM) (see Table 1 and Figure 3a). Exposure to EMS (500 µg/ml) was used a positive control throughout this study.

**Table 1.** Summary of relative cell counts(cytotoxicity) and %tail DNA genotoxicity data of validation materials for RICom assay. %tail DNA is the mean of three independent experimental replicate tissues (based on median %tail DNA per individual tissue).

Compound	Test concentration (µg/ml)	Test concentration (mM)	% Tail DNA		% Relative cell count	
			Mean	St. dev	Mean	St. dev
EMS	solvent	solvent	2.59	0.11	100.00	0.00
	50.0	0.40	3.46	0.83	101.33	10.06
	500	4.03	26.25	3.14	89.93	8.41
	1000	8.05	36.48	4.31	83.65	19.43
ENU	solvent	solvent	3.35	2.30	100.00	0.00
	73.19	0.63	13.03	1.36	91.52	7.41
	146.4	1.25	32.52	6.28	90.11	11.27
	292.8	2.50	52.83	2.26	65.58	10.34
	control	control	36.50	7.27	98.82	13.53
Phen HCl	solvent	solvent	2.96	1.24	100.00	0.00
	121	0.50	2.59	1.83	99.53	11.60
	242	1.00	2.67	1.27	101.30	4.57
	484	2.00	2.85	0.94	101.58	23.64
	control	control	32.91	5.62	77.28	25.35
B[a]P	solvent	solvent	4.02	1.03	100.00	0.00
	10.24	0.032	8.69	2.38	82.79	16.84
	12.8	0.040	11.86	2.18	62.60	6.19
	16	0.051	12.16	1.50	63.68	10.42
	20	0.063	12.52	0.24	66.37	8.49
	25	0.079	13.60	1.12	68.29	17.63
	control	control	34.85	4.86	70.94	18.83
DMH	solvent	solvent	3.10	2.90	100.00	0.00
	332.5	2.5	2.71	2.93	99.53	21.38
	665	5	2.52	2.41	101.30	5.60
	1330	10	2.47	2.31	101.58	6.32
	control	control	34.85	8.14	92.96	17.45
Etop	solvent	solvent	3.46	1.04	100.00	0.00
	0.5	0.0043	3.68	2.40	105.42	19.07
	5	0.0085	3.65	2.71	95.47	26.61
	10	0.017	3.74	2.25	79.97	9.19
	control	control	29.53	5.87	67.36	23.48
KBr	solvent	solvent	3.61	0.32	100.00	0.00
	520.6	3.09	13.13	3.50	86.69	2.63
	585.7	3.48	13.10	2.03	82.65	8.56
	658.9	3.91	13.19	3.73	71.40	0.03
	741.2	4.40	13.56	3.15	67.27	9.01
	control	control	36.85	2.36	69.55	7.36
GA	solvent	solvent	2.20	0.97	100.00	0.00
	13.7	0.16	6.59	1.30	88.59	8.25
	27.4	0.31	14.62	3.87	97.17	0.46
	54.9	0.63	32.22	7.36	95.14	8.57
	109.8	1.25	49.53	4.69	90.12	4.88
	control	control	36.85	2.36	68.22	9.43

EMS: Ethyl methanesulfonate. ENU: ethyl nitrosourea, Phen HCl: phenformin hydrochloride, B[a]P: benzo[a]pyrene, DMH- 1,2-dimethylhydrazine, Etop: etoposide, KBr: potassium bromate, GA:glycidamide.

### Ethylnitrosourea, ENU (CAS 759-73-9)

ENU is a potent direct-acting alkylating agent, which induces DNA damage such as base mutations, strand breaks, and alkali-labile lesions [39,40]. The maximum test concentration was limited to 292.8 µg/ml (2.5 mM). Concentrations above 2.5 mM did not yield analysable comet cells (i.e. all visible cells were hedgehogs). ENU induced a significant dose-dependent increase in %tail DNA in all test conditions, relative to the solvent control. The percentage induction was also above the historical negative values. This is evidence for a clear positive comet result and EpiIntestinal™ tissues are sensitive to ENU and respond as predicted for human intestinal epithelia *in vivo* (see Table 1 and Fig. 3b).

### Benzo[a]pyrene, BaP (CAS 50-32-8)

Smoke drying, or high-temperature food processing, may lead to deposition of BaP on food surfaces [41]. BaP is a pro-genotoxin requiring metabolism to generate its genotoxic epoxide metabolite; BaP is categorized as a group 1 carcinogen by International Agency for Research on Cancer [42]. BaP compounds can be metabolized by cytochrome P450 1A1 enzymes and epoxide hydrolases [43–47]. The final epoxide metabolite can intercalate with DNA and distorts the helical structure [48]. Some commonly used *in vitro* models in genotoxicity testing do not possess this metabolic capability and require supplementation with liver supernatant, such as S9 fraction [49]. Human small intestine is known to metabolise BaP *in vivo* [50]. The EpiIntestinal™ microtissues used in this study are verified to express the major intestinal phase I/II drug metabolizing enzymes such as CYP1A1, CYP3A4, UDP-glucuronosyltransferases and Sulfotransferase, and key efflux transporters including ABCB1/MDR1, as previously characterized in Ayehunie *et al*. [16] and Cui [51]. In our hands, the maximum test concentration was limited by the aqueous solubility of BaP at 20 µg/ml (79 µM). BaP was found to induce DNA damage in all treatment groups in EpiIntestinal™ tissues, albeit it was less potent than EMS and ENU (see Table 1 and Figs. 3c). Comet tails indicative of DNA damage were evident in EpiIntestinal™ tissue samples following BaP treatment, without any need for metabolic supplementation, or inhibitors of DNA repair. We interpret that this demonstrates the 3D EpiIntestinal™ tissues retain metabolic capacity to generate BaP genotoxicity recapitulating the response of native human small intestine.

### Phenformin hydrochloride, Phen HCl (CAS 834-28-6)

Phen HCl is a chemical agent belonging to the biguanide class and was chosen for this study as a candidate cytotoxic but non-genotoxic test material. Phen HCl is recommended by the European Centre for the Validation of Alternative Methods (ECVAM) for use in new and improved genotoxicity assays [52]. It is known to be well absorbed by the small intestine and causes intestinal side effects [53,54]. The maximum test concentration was chosen as 484 µg/ml (2 mM), above the recommended test concentration limits, but the authors desired to elicit maximal cytotoxicity responses in order to test the limits of the EpiIntestinal™ technology platform. Whilst a reduction in cell number was observed, we did not observe any change in % tail DNA above historical background measures at any tested concentration (see Table 1 and Fig. 3e).

### Dimethylhydrazine hydrochloride, DMH (CAS 306-37-6)

DMH is a potent carcinogen and a well-known positive control used commonly with *in vivo* liver and colon comet assays [5]. For our system, the maximum test concentration for DMH was limited to 1330 µg/ml (10 mM). At the end of treatment, we did not observe any decrease in cell number, we did not observe any statistically significant increase in % tail DNA, and we did not exceed the established historical negative range at any test concentration (see Table 1 and Fig. 3f). Published *in vivo* data have reported genotoxic activity of DMH in the liver and colon, though it is reported as likely negative in the stomach and other organs when administered orally [55,56]. DMH is a pro-genotoxin reliant on metabolism by the enzyme, CYP2E1, forming azoxymethane, and subsequent unstable methylazoxymethanol that rapidly degrades forming formaldehyde and DNA alkylating methyldiazonium ions [57,58]. CYP2E1 is highly expressed in the liver [59]; expression is lower in the colon [60] and comparatively minimal in the upper intestine if at all [61]. 3D EpiIntestinal™ tissues do not express the complete repertoire of cytochrome P450 enzymes (i.e. CYP2E1 in this case), but are closer in representation to the metabolic capability of the native small intestine. Hence, we interpret that DMH is not likely metabolized by EpiIntestinal™ tissue samples. Without the capacity to metabolise DMH to the intermediate methylazoxymethanol, and subsequent formaldehyde and methyldiazonium ions, DMH has no genotoxic effect in the reconstructed EpiIntestinal™ tissue platform. This outcome is not unexpected.

### Etoposide, Etop (CAS 33419-42-0)

Etoposide, a topoisomerase II inhibitor, causes double-strand breaks. It prevents religation of the DNA strand. Topoisomerase II is more strongly expressed in rapidly dividing cells and tissues such as skin, lymph nodes, and bone marrow [62]. The maximum test concentration was limited to 10 µg/ml (17 µM) due to poor aqueous solubility. We noted that EpiIntestinal™ tissue samples exposed to Etop exhibited a mild decrease (~20%) in the total cell population of samples when exposed to the highest test concentrations; however, the comet analysis (% tail DNA) did not exceed the established negative range (see Table 1 and Figs. 3d). Whilst the intestinal tract is self-renewing, the organisation is that proliferative cells reside in the lower crypts of Lieberkühn and the villi structure itself is composed of differentiated nonproliferating cells, primarily ­enterocytes. Cells divide from the lower crypts and migrate upwards along the villi structure (excluding Paneth cells). Due to proliferation being focused on a narrow population of cells, etoposide is unlikely to have an effect on the terminally differentiated cell population [63–65]. The brief duration of exposure (48 h) and limited cell division and expression of topoisomerase II over this time in the EpiIntestinal™ tissue model is likely to have protected the tissue construct from Etop toxicity. In a 3D liver model derived from HepaRG cells, a negative result for Etop was also seen and was attributed to their non-proliferative and differentiated status [66]. In the 3D skin models, etoposide was only seen as a clear positive result with supplementation with aphidicolin to inhibit DNA repair [21]. *In vivo*, a small but significant increase in DNA damage was seen in the comet assay in the distal intestine of rats with 48-h treatment at the highest test concentration of 50 mg/kg when applied by intraperitoneal injection [67].

### Potassium bromate, KBr (CAS 7758-01-2)

KBr is used as an improving agent in bread products in some countries [68], though banned in others. It can cause DNA strand breaks and mutagenesis with positive results in in vitro and *in vivo* genotoxicity assays, is a known animal carcinogen and IARC 2B is possibly carcinogenic to humans [69–73]. KBr is rapidly absorbed by the intestine and damages intestinal tissues, brush borders, and DNA [74]. It is a powerful oxidising agent and its capacity to oxidise DNA bases also makes it a good positive control for the enzyme-modified comet assay [26, 75].

In this assay concentrations above 741.2 µg/ml (4.4 mM) were excessively cytotoxic with significant cell loss and damage to the EpiIntestinal tissue. KBr induced a small but significant increase in %tail DNA in all test concentrations, relative to the solvent control. The percentage induction was also above the historical negative values. This is evidence for a positive comet result and EpiIntestinal™ tissues are sensitive to KBr and respond as predicted for human intestinal epithelia *in vivo* (see Table 1 and Fig. 3g).

### Glycidamide, GA (CAS 5694-00-8)

Glycidamide is an epoxide metabolite of the food contaminant acrylamide that causes DNA adducts and mutations. It yields positive results in bacterial, *in vitro* micronucleus, mutation, and comet assays, and *in vivo* mammalian genotoxicity assays [76], and is a clear carcinogen in rat studies [77]. Whilst the authors could not find published data for *in vivo* comet specifically in the upper intestine, positive data were available for multiple other organs and for tumour formation in the small intestine [78].

The maximum test concentration was limited to 109.8 µg/ml (1.25 mM) that showed a moderate decrease in cell number. Concentrations above this did not yield analysable comet cells (i.e. all visible cells were hedgehogs). Glycidamide induced a significant dose-dependent increase in %tail DNA in multiple test concentrations relative to the solvent control. The %induction was also above the historical negative values. This is evidence for a clear positive comet result and EpiIntestinal™ tissues are sensitive to DNA damage by GA (see Table 1 and Fig. 3h).

### Comet compatibility of the model and limitations

Based on our findings (see Table 2), the reconstructed 3D EpiIntestinal™ tissue platform demonstrates that it ­possesses potential as a tool for hazard identification as an intestinal model, with correct identification of 7 of 8 test materials. We have adapted validated OECD and published comet assay protocols, for reproducible use in the MatTek^®^ 3D human EpiIntestinal™ tissue platform, accepting that the EpiIntestinal model does have certain limitations. We demonstrated that universal recommended comet assay positive materials such as EMS and ENU induced substantial, reproducible, and statistically significant DNA damage that were detected through the RICom assay protocol. These challenge materials are well-known materials with established genotoxicity evident in both *in vitro* and *in vivo* assay protocols. Notably, the reconstructed 3D EpiIntestinal™ tissue model expresses a constitutively low background %tail DNA, and low coefficient of variation in dosed and solvent control groups. Additional test materials such as KBr and GA also generated conclusive positive results in the assay.

**Table 2.** List of tested materials, their mode of action and a comparison of expected results between comet in EpiIntestinal tissues and *in vivo* results

Test material name	Mode of action	Description/reason for use	EpiIntestinal comet	*In vivo* result	Conclusion
Ethyl methane sulfonate	Alkylating agent	Commonly used *in vivo comet* positive control, recommended for any tissue type	+ve	+ve	Correct result
Ethyl nitrosourea	Alkylating agent	Commonly used *in vivo* comet positive control, recommended for many tissue types including upper intestine	+ve	+ve	Correct result
Phenformin hydrochloride	Non-genotoxic, biguanide drug	Recommended for use as non-genotoxic validation material, well absorbed by small intestine. Can cause other intestinal side effects	−ve	−ve	Correct result
Benzo[a]Pyrene	Pro-genotoxin, DNA adduct formation	Commonly used pro-genotoxin positive control for genotoxicity assays, requires CYP1A1 metabolism. Food contaminant	+ve	+ve	Correct result, metabolism of B[a]P to genotoxic metabolite
1,2-Dimethylhydrazine	Pro-genotoxin, alklyating agent	Recommended positive control for *in vivo* comet in liver and intestine. Requires CYP2E1 metabolism	−ve	−ve, stomach negative. But liver/colon +ve	Correct result, native small intestine does not metabolise DMH, occurs predominantely in liver
Etoposide	Topisomerase II inhbitor	Recommended for use as genotoxic validation material, commonly used as positive control for *in vitro* genotoxicity assay	−ve	+ve	Incorrect result
Potassium bromate	Oxidiser.	Food production additive. Damages intestinal DNA	+ve	+ve	Correct result
Glycidamide	DNA adduct formation	Acrylamide metabolite, increased incidence of small intestine tumours in mice	+ve	+ve in other organs, upper intestine no data. Small intestine carcinogen positive in mice	Correct result

It was also observed that the 3D model has partial competency for xenobiotic metabolism as previously demonstrated [16, 51]. This was evident in samples challenged with BaP and DMH. Measurable responses were evident in the absence of co-incubation with liver supernatants (S9), a metabolic activator, or a DNA repair inhibitor, that may be required to elicit genotoxicity of BaP in popular *in vitro* assay protocols that use human lymphocytes or TK6 lymphoblasts. In the 3D EpiIntestinal™ model, BaP was readily metabolised and induced a clear positive response without needing additional exogenous metabolic activators (e.g. S9), or inhibition of DNA repair (e.g. aphidicolin (APC)).

DMH, a recommended positive test material for colonic DNA damage and colon cancer in rats, failed to illicit %tail DNA or cytotoxicity measures. *In vivo* DMH has been shown to preferentially cause tumorigenesis in the large intestine and colon, with limited data of tumours in the small intestine in rodents. Route of ­application is a crucial factor with differing outcomes amongst intraperitoneal, subcutaneous, and oral application [56, 58, 79]. There is also evidence of selectivity for rodent DNA damage in the colon but not in other sections of the gastrointestinal tract [80]. The genotoxic and carcinogenic effects of DMH are reliant upon metabolism in the liver where metabolites are then circulated into the intestinal tract via bile excretion and blood circulation as well as to other organs [81]. The mechanism of DMH genotoxicity is indirect; requiring CYP2E1 activity to generate metabolic intermediates and genotoxic metabolites. We interpret that this lack of response by the EpiIntestinal model likely reflects the restricted expression of CYP2E1 in mature 3D EpiIntestinal™ tissue samples. A standalone small intestinal model, such as EpiIntestinal™, is devoid of CYP2E1 expression mimicking that of native small intestinal tissue [61] and, consequently, DMH is not metabolised and is unable to effect its genotoxicity.

The human 3D EpiIntestinal™ model is a living, polarised epithelial tissue. In common with intestinal tissue *in vivo*, epithelial stem cells basal located in the basal compartment, proliferate, migrate apically, and differentiate. Once they have left the basal compartment, suprabasal cells no longer proliferate. We interpret that this is the reason exposure to Etoposide had little impact on EpiIntestinal™ tissue %tail DNA. This outcome stands in contrast with commonly used *in vitro* model systems, such as TK6, in which Etop induces clear evidence for genotoxicity, and the limited *in vivo* intestinal comet data available. We further note that Etop has yielded variable results as a test material in other 3D tissue models [15, 66].

In this report we present data that we interpret supports the utility of the human 3D EpiIntestinal™ tissue platform for use with the comet endpoint assay, we suggest that the 3D EpiIntestinal™ technology platform is suitable for further investigation as an intestinal *in vitro* assay for genotoxicity and for aiding mechanistic understanding of ingested materials. With greater authenticity than cell lines, it offers compatibility, tissue-specificity, and reliability prior to verification with *in vivo* studies.

### Future work

In addition to its authentic cellular organisation, architecture, and structure, the human 3D EpiIntestinal™ tissue platform recapitulates characteristic physiological aspects of human intestinal epithelium. This provides an opportunity for additional interrogation and enquiry. We noted that 3D EpiIntestinal™ tissue does, however, possess some limitations. Due to low proliferation in the epithelial stem cell population, cell cycle-dependent events are limited in comparison to existing protocols that utilise transformed cell lines. As the model includes differentiated intestinal cells, some metabolic and detoxification pathways are absent which in other protocols and culture models may be compensated through the use of S9 liver fractions. This limits the utility of the 3D EpiIntestinal™ platform when assaying compounds that require liver-specific metabolic activation such as DMH. To expand the assay repertoire supplementation with liver supernatant, S9 fraction, may expand the accessibility of the platform to embrace these important compounds. However, the authors also wish to specify that a proposed advantage of the model, and this is not specific to genotoxicity assessment, is its ability to understand human relevant mechanisms and better recapitulate the structure and metabolism of native human upper intestinal epithelia. The aim is not to recapitulate systemic and all encapsulating metabolism but rather that of the individual organ and better understand the mechanism of genotoxic action in the small intestine, a major site of initial contact for foodborne carcinogens.

The materials tested in the comet assay for this paper are well-characterized materials for genotoxicity assay validation. Although it is not possible to make a definitive statement about sensitivity or specificity with this initial limited dataset, our findings demonstrate the potential of the assay and model as a tool for assessing orally ingested materials. And it has long been recognised that many chemicals with rodent carcinogenic activity can be found in commonly consumed foods [82]. The intent of the authors is to further validate and expand the number of materials tested with this assay protocol, including testing mixtures and foods containing genotoxic materials, so as to determine the sensitivity and specificity of the EpiIntestinal model and demonstrate its applicability to testing ‘real life’ samples. And whilst the EpiIntestinal tissues are maybe more akin to that of native human intestine than other readily available models, this is also reflected in cost and this basic comet validation presented here does not yet show it improves upon other available models for assessment of genotoxicity. Further work is also required to justify the cost-benefit of using this model.

The work presented herein is for a relatively short-duration assay time of 48 hs. We acknowledge that human 3D EpiIntestinal™ tissue remains viable over longer periods. This represents opportunities to assay events that take place over longer durations. The sister micronucleus assay (RICyt) utilizes a 10-day treatment time due to the limited number of basal epithelial stem cells, and their slow cell cycle [31]. Future development of the human 3D EpiIntestinal™ tissue platform in the toxicology space might assay materials of concern over extended durations, durations that might affect outcmes such as oxidative stress [83], premature differentiation, hyperplasia [84], and dysbiosis [85]. Notwithstanding, the comet assay protocol is accessible for further modification, with inhibition of DNA repair, or enzymatic conversion of oxidized bases to strand breaks [75], which offers potential to increase sensitivity of the assay to additional types of DNA damage. Upcoming new models from the manufacturer of the EpiIntestinal model derived from colonic stem cells may offer organ specificity for the large intestine and could be combined with the RICom and RICyt assay protocols. We suggest that through the adoption of testing protocols that embrace 3D tissue models, the breadth and depth of toxicological analyses will be enhanced, yielding greater specificity, wider modes of action, and greater authenticity, thus relevance for human physiology.

## Data Availability

Post publication the data underlying this article will be shared on reasonable request to the corresponding author.
